# Inhibiting β-catenin disables nucleolar functions in triple-negative breast cancer

**DOI:** 10.1038/s41419-021-03531-z

**Published:** 2021-03-04

**Authors:** Shannon E. Weeks, Sarah C. Kammerud, Brandon J. Metge, Heba A. AlSheikh, David A. Schneider, Dongquan Chen, Shi Wei, James A. Mobley, Akinyemi I. Ojesina, Lalita A. Shevde, Rajeev S. Samant

**Affiliations:** 1 Department of Pathology, University of Alabama at Birmingham, Birmingham, AL USA; 2 Department of Biochemistry and Molecular Genetics, University of Alabama at Birmingham, Birmingham, AL USA; 3 O’Neal Comprehensive Cancer Center, University of Alabama at Birmingham, Birmingham, AL USA; 4 Department of Medicine, University of Alabama at Birmingham, Birmingham, AL USA; 5 Department of Anesthesiology and Perioperative Medicine, University of Alabama at Birmingham, Birmingham, AL USA; 6 Department of Epidemiology, University of Alabama at Birmingham, Birmingham, AL USA; 7 Birmingham VA Medical Center, Birmingham, AL USA

**Keywords:** Breast cancer, Organelles, Breast cancer, Metastasis

## Abstract

Triple-negative breast cancer (TNBC) patients with upregulated Wnt/β-catenin signaling often have poor clinical prognoses. During pathological examinations of breast cancer sections stained for β-catenin, we made the serendipitous observation that relative to non-TNBC, specimens from TNBC patients have a greater abundance of nucleoli. There was a remarkable direct relationship between nuclear β-catenin and greater numbers of nucleoli in TNBC tissues. These surprising observations spurred our investigations to decipher the differential functional relevance of the nucleolus in TNBC versus non-TNBC cells. Comparative nucleolar proteomics revealed that the majority of the nucleolar proteins in TNBC cells were potential targets of β-catenin signaling. Next, we undertook an analysis of the nucleolar proteome in TNBC cells in response to β-catenin inhibition. This effort revealed that a vital component of pre-rRNA processing, LAS1 like ribosome biogenesis factor (LAS1L) was significantly decreased in the nucleoli of β-catenin inhibited TNBC cells. Here we demonstrate that LAS1L protein expression is significantly elevated in TNBC patients, and it functionally is important for mammary tumor growth in xenograft models and enables invasive attributes. Our observations highlight a novel function for β-catenin in orchestrating nucleolar activity in TNBCs.

## Introduction

The nucleolus is a non-membrane-bound, subnuclear body that is known primarily for its role in ribosome biogenesis. The nucleolus forms around nucleolar organizer regions (NORs). It is composed of three distinct sub-compartments, the fibrillar center, the dense fibrillar component, and the granular component, each of which is responsible for different aspects of ribosome biogenesis. Ribosomes are the macromolecular ensembles of ribosomal RNA (rRNA) and ribosomal proteins. Ribosomal DNA (rDNA) codes for rRNA and is located as tandem-repeats on five acrocentric chromosomes (chromosomes #13,14,15, 21, and 22). The transcription of rDNA is exclusively accomplished by RNA polymerase I (RNA Pol I)^1,2^. Several hundred proteins are involved in ribosome biogenesis, and thus, the nucleolus is a huge depot of proteins. Despite being recognized primarily for its role in ribosome biogenesis, evidence suggests that the nucleolus plays critical roles in both normal biologies of the cell, as well as pathological states, including cancer^3^. More abundant and hyperplastic nucleoli were first recognized as hallmarks of cancer in the late 1800s by an Italian pathologist, Giuseppe Pianese, and thereafter were used by pathologists as a prognostic indicator of cancerous lesions^4,5^. Increased nucleolar size and number are classically attributed to the increased need for protein synthesis in cancer cells so that they can maintain their rapid rate of division^6^. Advances in proteomics and microscopic techniques have revealed that the nucleolus has a complex and dynamic proteome^7,8^. Thus, based on cell type and its physiologic state, the nucleolar proteome of a cell shows variations. It is advocated that understanding the composition of the nucleolar proteome is central to revealing cues that are of the utmost importance to understanding its functional contributions to normal and cancer biology^9^.

Breast cancer is the most frequently diagnosed cancer in women in the US and is the second leading cause of cancer-related deaths in women worldwide. Breast cancer is broadly classified into four molecular subtypes based on hormone and growth factor receptor status; luminal A, luminal B, Her2 enriched, and triple negative^10^. Of the various subtypes of breast cancer, triple-negative breast cancer (TNBC) is known to be the most aggressive subtype that presents clinical challenges due to its rapid progression, drug resistance, metastatic relapse, and poor outcome^11,12^. TNBC patients with upregulated Wnt signaling often have a poor prognosis^12^. The ultimate effector of canonical Wnt signaling is TCF/LEF transcription co-factor, β-catenin. However, the effects of β-catenin signaling on the biology of the nucleolus and the resultant impact on TNBC progression remain unclear. Here we present our investigations of differences between TNBC and non-TNBC nucleolar proteomes that uncover a relationship between β-catenin signaling and ribosome biogenesis. To understand the contribution of β-catenin signaling, we investigated the impact of β-catenin inhibition on the proteome of TNBC nucleoli. We identified and confirmed that upon β-catenin inhibition the exclusively nucleolar protein, LAS1 Like, Ribosome Biogenesis Factor (LAS1L) is significantly decreased in TNBC nucleoli. Our studies identify the involvement of LAS1L in facilitating the rapid growth and metastatic ability of TNBC tumors.

## Materials and methods

### Cell lines and reagents

MDA-MB-468 cells (ATCC# HTB-132) were cultured in DMEM/nutrient mixture F-12 (DMEM-F12, Gibco) supplemented with 10% fetal bovine serum (FBS) (Gibco). SUM1315 cells (Asterand Bioscience, Detroit, MI) were cultured in DMEM-F12, supplemented with 10 µg/mL insulin (Sigma) and 20 ng/mL epidermal growth factor (EGF; Sigma). T47D cells (ATCC# HTB-133) were cultured in RPMI (Gibco) supplemented with 10% FBS and 10 µg/mL insulin. MDA-MB 231 cells (ATCC# HTB-26) were cultured in DMEM-F12 supplemented with 5% FBS. MCF7 cells (ATCC# HTB-22) and BT549 cells (ATCC# HTB-122) were cultured in DMEM-F12 media supplemented with 10 µg/mL insulin and 10% FBS. SUM159 cells (Asterand) and SUM149 (Asterand) cells were cultured in DMEM-F12 media supplemented with 10 µg/mL insulin, 1ug/mL hydrocortisone (Sigma), and 5% FBS. SkBr3 cells (ATCC# HTB-30) were cultured in McCoy’s 5A media (Sigma Aldrich) with 10% FBS. BT474 cells (ATCC# HTB-20EMT) were cultured in DMEM-F12 supplemented with 10% FBS. HCC1569 cells (ATCC# CRL-2330) were cultured in RPMI with 10% FBS. All cells were cultured at 37 °C in a humidified incubator with 5% CO_2_. Cell lines are routinely verified to be mycoplasma-free.

For all iCRT14 treatments, cells were treated with 5 µM iCRT14 (Tocris Bioscience) for 48 h.

### HC11 differentiation

HC11 cells were obtained from ATCC (CRL-3062) and maintained in RPMI 1640 with 10% FBS, 20 ng/ml EGF, and 5 μg /mL insulin. To differentiate HC11 cells, EGF was removed from the media 24 h prior to the addition of DIP (RPMI 1640 media containing 100 nM dexamethasone (Tocris)), 5 μg/mL insulin, and 5 μg/mL ovine prolactin (NIDDK-oPRL-21 was obtained from Dr. A.F. Parlow at the National Hormone & Pituitary Program, Harbor-UCLA Medical Center, Torrance, CA). DIP media was changed every 24 h, and the differentiation process was complete by 72 h. For iCRT14 treatment, undifferentiated HC11 cells were treated with 5 µM iCRT14 (Tocris) for 48 h. Levels of β-casein (Santa Cruz sc-17971 1:1000), total β-catenin (Cell Signaling #9582 1:5000), and phospho- β-catenin (Cell Signaling #5651 1:1000) were assessed by immunoblot. β-actin (Sigma A3854 1:10,000) was used as a loading control.

For AgNOR staining, HC11 cells were grown 60–70% confluent on a glass slide in a 60 mm dish, media was removed, and the cells were washed with PBS twice then fixed with 4%PFA for 15 min then washed twice. Mixed 1 part of 2% gelatin formic acid solution and 2 parts 50% aqueous silver nitrate solution and immediately added to the slides for 12 min at room temperature. The slides were washed followed by 10 min incubation in 5% sodium thiosulfate solution, then washed and mounted with DAPI (Vector lab H-1200). Images were captured using a Nikon Inverted Fluorescence Phase Contrast Microscope (TI-FL 546439) with 40X lens and analyzed using NIS-Elements BR 5.20.02 software.

*T*-test was applied for statistical analysis, using GraphPad Prism version 8 (GraphPad Software, La Jolla, CA). Nucleolar counts were deemed as outliers by GraphPad Prism were not included. Usually this corresponded with random cells with the illogical number of nucleoli. Comparisons were considered statistically significant for *p*-value < 0.05. Experiments were repeated once.

### Immunohistochemical staining

Immunohistochemical staining of LAS1L was performed using the Dako EnVision+ System (Agilent, Santa Clara, CA). Briefly, samples were deparaffinized and rehydrated before being subjected to heat-induced epitope retrieval by boiling in sodium citrate buffer. Samples were blocked with Dako Dual Endogenous Enzyme Block, then primary antibody (LAS1L HPA061463 or β-catenin HPA029160, both Sigma) was applied and samples were incubated overnight at 4 °C. After washing, samples were processed as per the manufacturer’s protocol. Staining intensity was scored on a scale of 0 (no staining) to 4 (strongest possible intensity). The percentage of cells staining at each intensity was determined. The percent of cells at each intensity was multiplied by the corresponding intensity value and the addition of the products was divided by 100 to get the immunoscore for each field^13^. Cytoplasm and nuclei were scored separately for each case. A total of eight fields were scored per case. The same 48 TNBC and 37 non-TNBC cases were scored for both targets.

### Nucleolar fractionation verification

Western blotting was used to validate purity of nucleolar fractions. Protein samples were separated using SDS-PAGE. The resolved proteins were transferred to a PVDF membrane. The membrane was blocked with 5% milk in TBST with 1% Tween at room temperature for 1 h, followed by incubation with primary antibody at 4 °C overnight. Following incubation with appropriate secondary antibody, the signal was visualized using ECL Prime or ECL Select (Amersham) using Amersham Imager 600.

The following antibodies were used: Fibrillarin (AbCam ab166630 1:1000), and Alpha Tubulin (Cell Signaling Technology #12351 1:1000).

### Transwell invasion assay

Cells were seeded in serum-free growth medium in BioCoat™ Matrigel® Invasion Chambers (Corning, Bedford, MA). A serum-free growth medium with fibronectin was added to each well containing the chamber insert. Cell-containing inserts were incubated in a 5% CO_2_ incubator at 37 °C for 16 h, then fixed with 4% paraformaldehyde, stained with crystal violet for 10 min, and rinsed with deionized water. Photographs of inserts were taken using Nikon Elipse E200LED MV R (Tokyo Japan) using a 10× objective. Each experimental condition was performed in duplicate and four random fields of each insert were recorded. Significance was determined using a *t*-test and all error bars indicate SEM.

### Migration assay

Cell culture insert filters (8 µM pore-size) were coated with 6 ng/ml of gelatin. Cells were plated in the inner chamber in serum-free media and placed in wells with 10% serum-containing media. The cells were incubated at 37 °C for 4 h then fixed with 4% paraformaldehyde, stained with crystal violet. Photographs of inserts were taken using Nikon Elipse E200LED MV R (Tokyo Japan) using a 10× objective. Each experimental condition was performed in duplicate and four random fields of each insert were recorded. Significance was determined using a *t*-test and all error bars indicate SEM.

### Chromatin immunoprecipitation (Chip)

Cells were plated on 10 cm dishes at 2.5 × 10^6^ cells per plate and the following day cells were treated with 10 mM Lithium Chloride. 24 h post-treatment; cells were processed using the Simple Chip Plus Enzymatic kit (Cell Signaling Technologies) as per the manufacturer’s protocol. Briefly, cells were fixed with 1% formaldehyde at room temp and cell pellets were processed for nuclei isolation and chromatin digestion with micrococcal nuclease and then sonicated. In total, 10 µg of cross-linked chromatin was immunoprecipitated with 20 µl anti-β-catenin antibody (Cell Signaling Technologies #8480). Chromatin was then eluted from the IP and cross-links were reversed followed by column purification of DNA. Purified DNA from Chip and input were subjected to real-time quantitative PCR to quantitate the amount of DNA associated with β-catenin in the LAS1L promoter sequence. PCR was done using 2X Maxima SYBR Green Master Mix (Thermo Scientific) along with primer pairs to amplify specific regions of the LAS1L promoter. Primer pairs used were as follows: β-catenin sites in LAS1L promoter For- CTT CCG GTC TGG TAC AGA AG Rev- CCA ATC GGA ACG TCA GAA TTG. *C*_*T*_ values of input DNA was used to calculate percent immunoprecipitation utilizing the following calculation: Percent of input = 2% × 2^(C[T] 2% Input Sample-C[T] IP Sample)^, values are represented as signal relative to input with beads signal subtracted from the specific pulldown. Each reaction was done in triplicate using an Applied Biosystems Step One Plus. Experiment was repeated one time as a biological repeat.

### Polysome profiling

Cells were treated with cycloheximide (100 mg/ml) 15 min prior to harvesting, washed three times in PBS (4 °C) containing 100 mg/mL cycloheximide, and scraped in PBS/cycloheximide. After centrifugation at 3000 rpm for 2 min at 4 °C, cells were lysed in hypotonic lysis buffer (5 mM Tris-HCL [pH7.5]; 2.5 mM MgCl_2_; 1.5 mM KCl). The lysates were centrifuged at 13,000 rpm for 5 min at 4 °C, and the supernatants were loaded onto a 10–50% sucrose gradient. The gradients were centrifuged in a Beckman SW40Ti rotor at 35,000 rpm for 3 h at 4 °C. Gradients were fractionated by pumping 80% sucrose into the bottom of each column and monitoring eluted fractions at 254 nm.

### Tumor growth

Examination of tumor growth was done as described previously^14^. Cells (1E6) suspended in HBSS were injected into the mammary fat pad of eight-week-old female NOD SCID mice. Six mice per group were used and the experiment was repeated one time with the independent passage of cells. Investigators were not blinded to any groups. Tumor growth was monitored by orthogonal measurements using Vernier caliper at a 3-day interval. Statistical significance was determined by two-way ANOVA followed by Dunnett’s post hoc test for tumor growth at respective time points and error bars represent SEM. Tumors were removed by survival surgery when control tumors reached a mean tumor diameter of 10 mm. Resected tumors were formalin-fixed paraffin-embedded. Eight weeks after tumor removal, the mice were euthanized, and the lungs were evaluated for metastasis. Lung metastases were enumerated using a Nikon StereoZoom microscope. The animal studies have been conducted in accordance with the approval and guidelines of the Institutional Animal Care and Use Committee (IACUC) of The University of Alabama at Birmingham.

### AgNOR staining

Human breast cancer arrays (BR1202a and BR1901; US Biomax, Rockville, MD) were used for AgNOR staining. Additional de-identified specimens were procured under IRB#00000726. In addition, mouse tumor sections were AgNOR-stained. Slides were deparaffinized and rehydrated before incubation in the staining solution (one-part 2% gelatin, 1% formic acid solution, and two parts 50% silver nitrate in water, at room temperature). Images were taken using Nikon Eclipse E200LED MV R (Nikon, Tokyo, Japan) at 40X. In total, nucleoli were quantified in 68 TNBC and 34 non-TNBC samples. At least 40 cells were counted per field and at least eight random fields were counted per specimen. Results were represented as the percentage of cells containing 1, 2, or 3+ nucleoli per cell. For the mouse samples, a total of five fields was counted for each of three samples in each of the three groups. Investigators were single-blinded to ensure data integrity. Significance was determined using a *t*-test and all error bars indicate SEM.

### Nucleolar staining and live-cell imaging

The stain was prepared by mixing 1 mL of PBS with 2 µL of Nucleolar-ID green (Enzo) and 3 drops of NucBlue Live Cell Stain ReadyProbes reagent (Invitrogen). Cells cultured in 35 mm plates at 80% confluence were washed once with PBS (37 °C). A mixture of 250 μL of stain mix and 250 μL of complete media was added to each plate. Plates were incubated at 37 °C in a humidified incubator with 5% CO_2_ for 15 min. Live cell nucleolar images were captured using a Nikon Eclipse Ti inverted microscope at 40× (Nikon). Eight fields were counted for each cell line. Experiments were repeated at least three times. Significance was determined using a *t*-test and all error bars indicate SEM.

### Nucleolar isolation

Nucleoli were isolated as described previously^15^. Briefly, cells were cultured in complete media. At 90% confluence, cells were washed three times with cold PBS, pH7.4, and scraped off the plate with a minimal volume of PBS. The pooled cells were centrifuged at 500×*g* for 5 min. The reference volume (RV) was then determined by visually estimating the volume of the cell pellet. Cells were then resuspended in 15 RV of Nucleoli Standard Buffer (NSB) 10 mM Tris-HCl, pH 7.4, 10 mM NaCl, 1 mM MgCl_2_, and 1X Halt protease and phosphatase single-use inhibitor cocktail (Thermo Fisher) and incubated on ice for 30 min. NP-40 (10%) was added to the cells to obtain a final concentration of 0.3% NP-40 (Roche Applied Science, Mannheim, Germany) and the cells were homogenized 30–50 times using a tight Dounce homogenizer (Wheaton 7 mL Dounce tissue grinder). The homogenate was centrifuged at 1200×*g* for 10 min and resuspended in 10 RV of 250 mM sucrose containing 10 mM MgCl_2_. The supernatant containing the cytoplasmic fraction was removed for future analyses. Nuclei were then purified from the homogenate by centrifugation at 1200×*g* for 10 min through an 880 mM sucrose cushion containing 5 mM MgCl_2_. Purified nuclei were resuspended in 10 RV of 340 mM sucrose containing 5 mM MgCl_2_ and sonicated using 10–25 bursts (depending on cell line) of 10 s with 1 min rest on ice between bursts. Phase-contrast microscopy was used to ensure no intact cells remained upon sonication and that the nucleoli were devoid of associated debris. Nucleoli were then purified from the homogenate by centrifugation at 2000×*g* for 20 min at 4 °C through an 880 mM sucrose cushion containing 5 mM MgCl_2_. Purified nucleoli were resuspended in 340 mM sucrose with 1× Halt inhibitor cocktail^TM^ (Thermo Fisher) and stored at −80 °C for future analysis. Alternatively, the nucleolar pellet was resuspended in PBS with a 1X Halt inhibitor cocktail for mass spectrometry analysis.

### Proteomics analysis

Proteomic analysis of nucleolar fractions was done using LC-MS. Samples were eluted in 1× final LDS sample buffer at 96 °C for 10 min. The eluate was collected on a magnetic stand, reduced, and denatured further at 70 °C for 10 min. The whole sample was loaded onto a 10% Bis-tris gel and stained overnight with Colloidal Coomassie. Each sample lane was digested with trypsin overnight in six fractions prior to LCMS analysis. Data were analyzed using Scaffold 4 (information taken from LIMS report). For stringent analysis, protein threshold was set to 99%, minimum two peptide match, and peptide threshold 80%. The scaffold was used to identify proteins that were significantly different between the TNBC and Non-TNBC nucleolar proteomes. The experiment was done in duplicate. The proteomics data are deposited at ProteomeXchange via the PRIDE database. All nucleolar proteomics were done in independent biologic repeats. The data is deposited as two different submissions in ProteomeXchange via the PRIDE database. The details are below.

Submission details:

Project Name: Differential analysis of nucleolar proteomics in triple negative breast cancer

#1 Project accession: PXD021201 Project DOI: 10.6019/PXD021201

#2 Project accession: PXD021181 Project DOI: 10.6019/PXD021181.

### Immunocytochemistry

SUM 1315 cells (1 × 10^5^) were plated on FluoroDishes (World Precision Instruments, Sarasota, FL) and incubated overnight. Cells were fixed in 4% formaldehyde diluted with 1X PBS for 15 min at room temperature, then washed three times for 5 min with 1X PBS. Cells were then blocked for 1 h using 5% goat serum in PBS containing 0.3% Triton™ X-100. Primary antibodies were diluted 1:500 in 1% BSA in PBS containing 0.3% Triton™ X-100. (Fibrillarin Abcam (38F3) Ms mAb Ab4566) (LAS1L Sigma Rb Polyclonal HAP061463) and incubated for 2 h at room temperature. A secondary antibody with fluorochrome conjugation was diluted 1:1000 in 1% BSA in PBS containing 0.3% Triton™ X-100 and incubated at room temperature for 1 h. Cells were then sealed with VECTASHIELD with dapi (Vector Laboratories) (and sealed with nail polish). Images were captured with Nikon Eclipse Ti-U using 40X magnification.

### Luciferase assay

The responsiveness of the predicted TCF consensus sequence in LAS1L was assessed by utilizing a luciferase reporter assay. Consensus TCF site upstream of Las1L transcription start site (−139 bp) was synthesized and cloned into pGL3 promoter vector. Cells (10,000 per well) were transfected using Lipofectamine 2000 as per the manufacturer’s protocol. Cells were treated with 5 µM iCRT or control (DMSO) 24 h after transfection. Luciferase activity was determined using dual-luciferase reporter (DLR) Assay Systems (Promega, Madison, WI). Relative luciferase units were normalized to total protein.

### Statistical analysis

Prism 8 (GraphPad) was used for data visualization and statistical analyses. All data were statistically analyzed using Student’s *t*-test (two-tailed) or one-way ANOVA with Bonferroni’s multiple comparison test. A *p-*value of < 0.05 was deemed significant for all analyses. The results shown are representative examples from at least three independent replicates (unless stated differently). All error bars shown represent the standard error of the mean. Statistical significance was defined as *P* < 0.05. Details about specific tests applied are in the respective legends. Unless otherwise noted below, statistics were calculated from *n* = 3 technical replicates from an individual experiment. Variance is similar across the groups. Data are reported as means ± standard deviations (unless otherwise specified).

## Results

### TNBC cells have more nucleoli per nucleus compared to non-TNBC cells

**Fig. 1:**
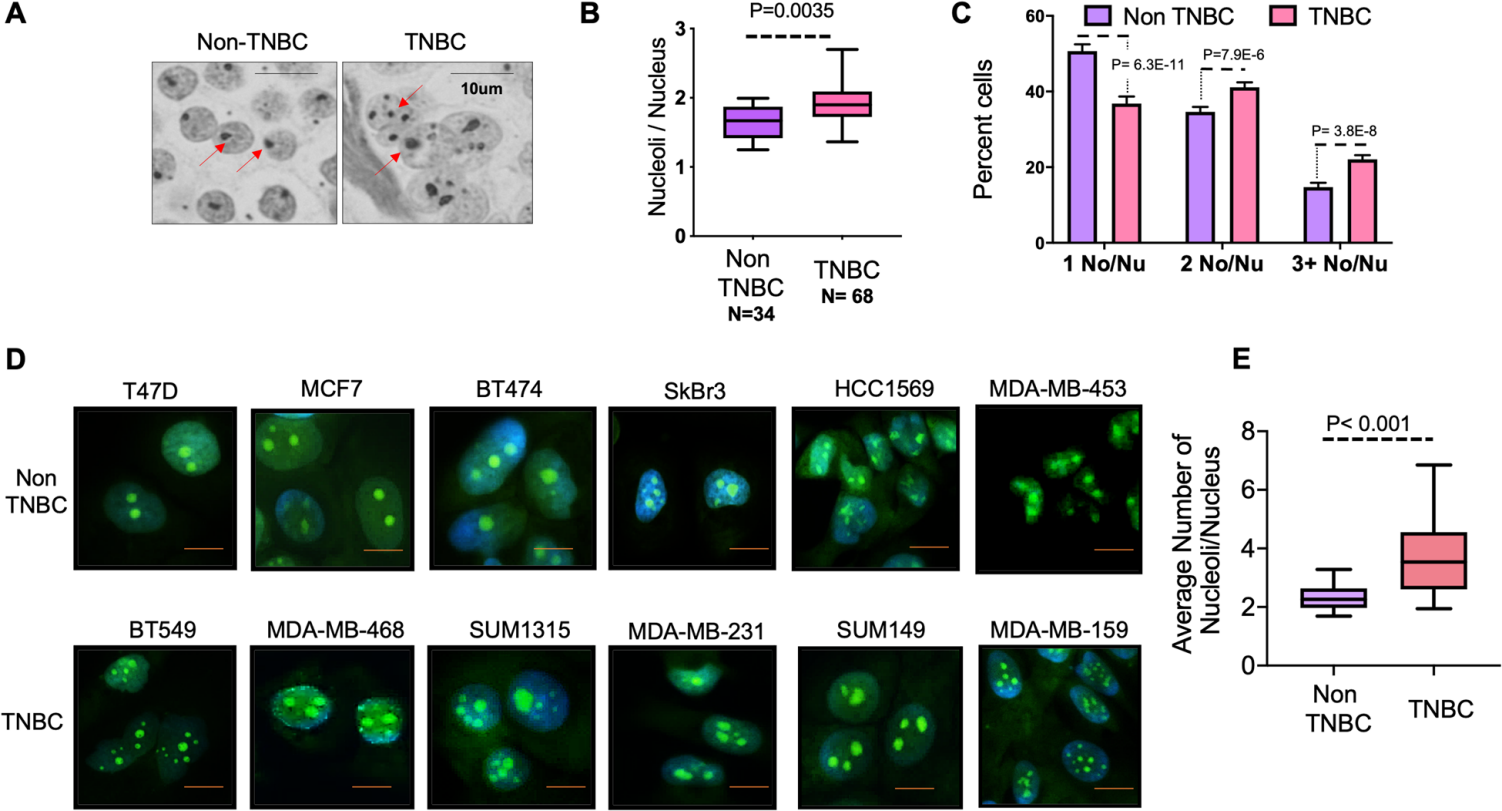
TNBC cells have more nucleoli than non-TNBC cells. **A** Representative image of AgNOR stained human breast cancer tissue sections classified as TNBC or non-TNBC. Red arrows point at nucleoli. **B** Quantification of nucleolar number (using AgNOR staining) in human breast cancer tumor tissue sections classified as TNBC or Non-TNBC. Number of nucleoli per nucleus from cells in each specimen were counted. The data is represented as a box and whiskers plot. **C** Cells from each human breast cancer tumor tissue section, classified as TNBC or non-TNBC were separated into groups of cells with one nucleolus/nucleus, two nucleoli/nucleus, and three or more nucleoli/nucleus. These groups were represented as percent cells in each group. **D** Representative images of NucleolarID^TM^ stained cells from TNBC or Non-TNBC cell lines. Focused bright green staining demarcates nucleoli. DAPI staining (blue) marks the nucleus. **E** Quantification of nucleolar number (using NucleolarID^TM^ staining) in human breast cancer cell lines. The number of nucleoli per nucleus from six TNBC and six non-TNBC cell lines was counted. The data is represented as a box and whiskers plot and was significantly decreased in the non-TNBC cell lines (*P* < 0.001). Significance was determined using a *T*-test and all error bars indicate SEM.

Classical pathologic diagnosis of tumor tissue has revealed that an increased number of nucleoli and/or nucleolar hypertrophy are predictive and prognostic parameters of increased mortality^16,17^. About 15–20% of breast cancers are triple-negative. Compared to other subtypes, TNBC is characterized by higher rates of relapse, greater metastatic potential, and overall poor survival^11^. We evaluated a cohort of clinical specimens from TNBCs and non-TNBCs to investigate if nucleoli present a distinguishing feature for TNBCs. AgNOR staining, a silver (Ag) based stain for identification of argyrophilic NOR serves as a clear contrasting tool to enumerate nucleoli^18,19^. We evaluated breast cancer tissue microarrays using AgNOR staining (Fig. 1A). Patient tissue classified as triple-negative had significantly greater numbers (*p* = 0.035) of nucleoli per nucleus in comparison with non-TNBC specimens (Fig. 1B). In tumor tissue, individual tumor cells may be in various physiologic states, and thus there is an inherent heterogeneity in nucleolar number per cell within an individual specimen. We stratified the AgNOR counts from patient specimens into three groups: cells with one nucleolus/nucleus, two nucleoli/nucleus, and cells with three or more nucleoli/nucleus. We observed that over 50% of cells from non-TNBC specimens showed a single nucleolus/nucleus, whereas about 65% of cells from TNBC specimens showed two or more nucleoli per nucleus (Fig. 1C). In order to examine if this characteristic increase in nucleolar number is also reflected in human breast cancer cell lines, we analyzed the nucleolar numbers from six TNBC and six non-TNBC cell lines using NucleolarID stain (Fig. 1D). We found that TNBC cell lines had significantly (*p* < 0.001) more nucleoli per nucleus than non-TNBC cell lines (Fig. 1E).

### TNBC cells have distinct nucleolar contents compared to non-TNBC cells

**Fig. 2:**
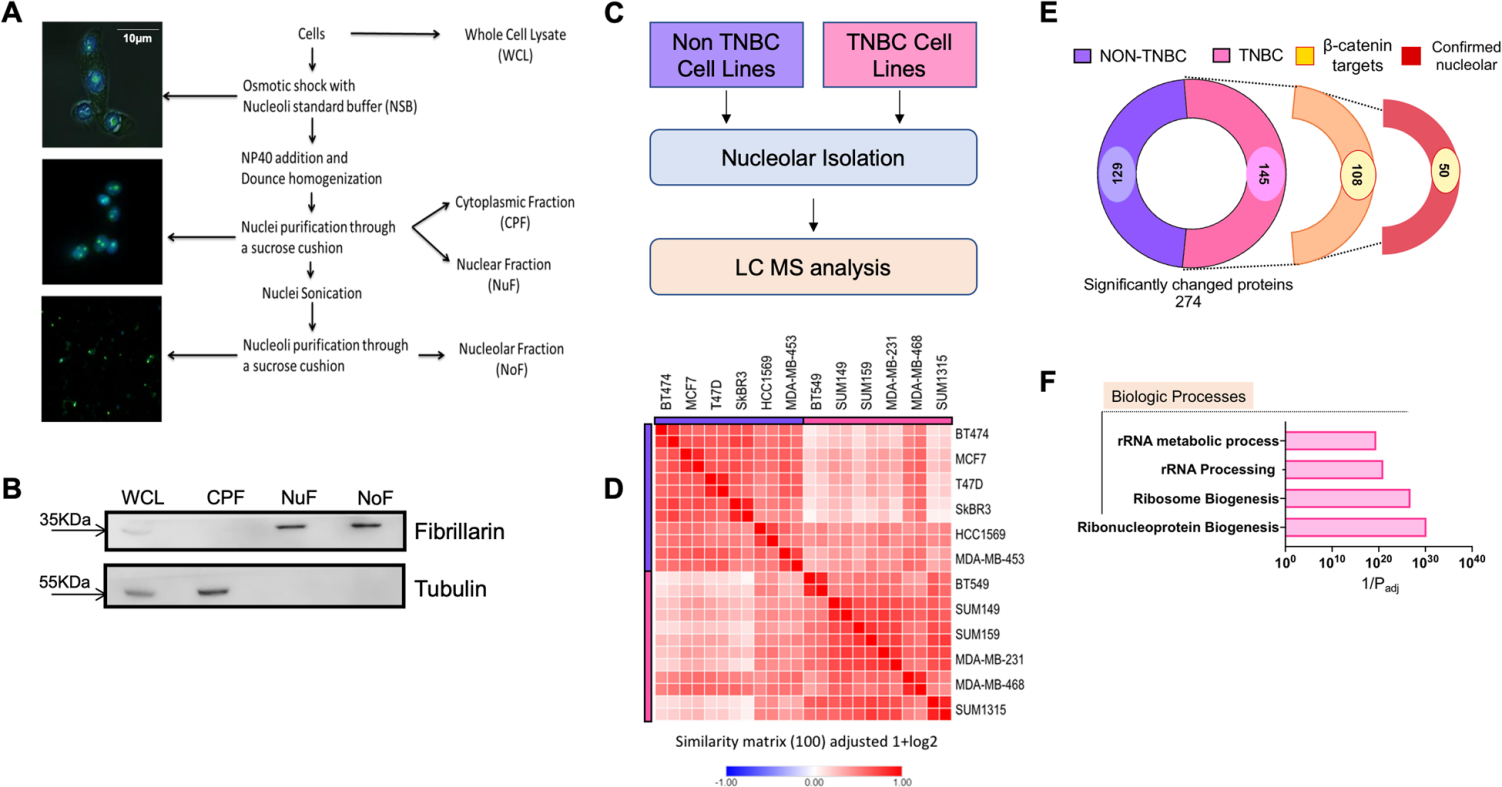
Identification of differences in nucleolar proteomes of TNBC and non-TNBC cells. **A** Graphical representation of the steps involved in isolating nucleoli from breast cancer cells. **B** Representative immunoblot showing verification of enrichment of nucleolar proteins. Fibrillarin is used as an indicator of nucleolar proteins. The absence of tubulin from the nuclear and nucleolar fractions was monitored to ensure purity. **C** Schematic summarizing workflow for nucleolar isolation and proteomic analysis to identify significantly altered proteins in the nucleolus between TNBC and non-TNBC cell lines. **D** Similarity matrix comparing nucleolar proteomes of TNBC cell lines with that of Non-TNBC cell lines. The proteomes were determined from two biological replicates. The matrix shows proteomes from both replicates of each cell line. **E** Graphical break-down of significantly altered proteins found in the nucleolar proteomics of the TNBC groups. Out of 145 proteins enriched in TNBC nucleoli, 108 were potential transcriptional targets of β-catenin. Out of these, 50 proteins were already reported to be nucleolar in one or more previously reported nucleolar proteomes from studies conducted in other (not breast cancer) cell types^8,28,29^. **F** Gene ontology analysis of proteins enriched in the nucleolar proteome of TNBC compared to Non-TNBC for biologic processes. The analysis was performed using g:Profiler™^30^.

Due to the dynamic nature of the proteome^7,20,21^, we hypothesized that the differences in TNBC nucleoli are not restricted to nucleolar numbers alone, but also seen in the protein contents of the nucleoli. Thus, nucleoli from each breast cancer cell line were isolated using the nucleolar isolation scheme illustrated in (Fig. 2A). The experiment was repeated using an independent passage of each cell line for ensuring the rigor and consistency of the data. The enrichment of nucleoli was always confirmed using immunoblotting. A representative blot is depicted in Fig. 2B. Fibrillarin is an exclusively nucleolar protein and is widely used as a marker of nucleoli, thus its presence endorsed the nuclear fraction (NuF) as well as the nucleolar fraction (NoF)^22^. Alpha tubulin is an exclusively cytoplasmic protein, and its absence evidenced a lack of cytoplasmic contamination in isolated nucleoli. These nucleolar fractions were subsequently evaluated for changes in proteomic content using mass spectrometry analysis. For a graphical representation of workflow see Fig. 2C. Similarity matrix determination carried out using Morpheus© showed that the proteomic contents of all nucleoli had a noteworthy similarity (Fig. 2D)^23^. This is expected as these cell lines are all from the same tissue of origin. While the nucleolar proteomes of TNBC cells cluster together and overlap as a group, collectively their nucleolar proteome is more distinct than that of the non-TNBC cells (Fig. 2D).

Using Scaffold^TM^ software, we determined that there were 274 proteins with significantly altered abundance between the nucleoli of the two groups. A heatmap of the 100 most significantly abundant proteins in TNBC cells and 100 most significantly abundant proteins in non-TNBC cells shows the extent and consistency of enrichment of individual proteins across various cell lines (Supplementary Fig. 1). Nucleoli of TNBC showed enrichment of 145 of these 274 proteins (Fig. 2E and Supplementary Table I).

β-catenin activation is noticeably increased in TNBCs and predicts poor outcome^24,25^. In fact, nuclear accumulation of β-catenin correlates with high levels of a CD44^high^/CD24^low^ stem cell population in TNBCs, which is characteristic of poor outcome^25,26^. Thus, we checked if the 145 proteins enriched in the nucleoli of TNBC cells were possible targets of Wnt/β-catenin signaling. Using ENCODE ChIP-seq data^27^, we found that 108 out of these 145 proteins could be potential targets of TCF/LEF signaling (Supplementary Table II). These proteins were compared across available nucleolar proteomes from the human protein atlas (HPA) as well as two published nucleolar proteomes by Ahmad et al. and Jarboui et al.^8,28,29^. We found that 50 proteins of these 108 proteins were also present in one or multiple of the published nucleolar proteomes. This sub-group of TNBC enriched TCF/LEF signaling target nucleolar proteins are referred to here as “confirmed” nucleolar proteins (Fig. 2E).

We then performed functional enrichment analysis of the 108 proteins predicted to be the targets of TCF/LEF signaling using g:Profiler™^30^. Based on ontology terms for biologic processes, these proteins were enriched for the term ribonucleoprotein biogenesis, ribosome biogenesis, rRNA processing, and rRNA metabolism (Fig. 2F).

### Inhibition of Wnt signaling leads to a reduction in nucleolar number in TNBC

**Fig. 3:**
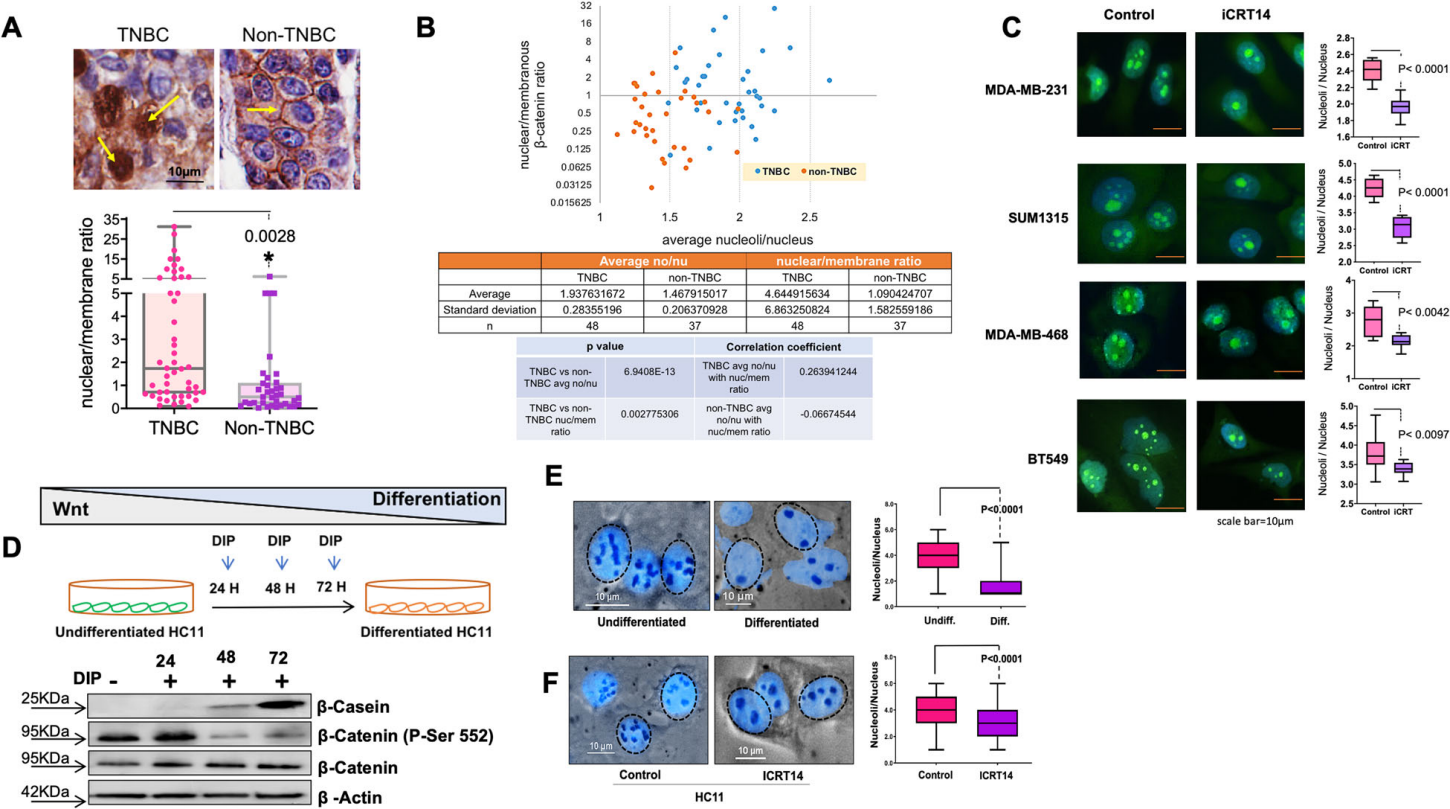
Active β-catenin signaling in TNBC contributes to the increase in nucleolar number. **A** Patient-derived breast cancer specimen was stained for β-catenin illustrating a reduction in nuclear to the membranous ratio of β-catenin indicating a reduction in active β-catenin signaling in non-TNBC patient tumor samples compared to TNBC patient tumor samples. Representative pictures of immunohistochemical staining for β-catenin in TNBC and non-TNBC specimens. Yellow arrows indicate intense staining locations for β-catenin. The staining was scored for nuclear β-catenin staining as well as membranous β-catenin staining. The ratio of nuclear vs. membranous β-catenin is depicted in the box and whiskers plot. Each dot indicates the ratios corresponding to each patient specimen. Statistical analysis was performed with a *T*-test and error bars indicate SEM. **B** A comparison of the ratio of nuclear vs. membranous β-catenin staining against the nucleolar number of each patient-derived specimen indicates the correlation between active Wnt signaling, nucleolar number, and breast cancer subtype. The tables below the plot summarize the details of specimens and statics. **C** TNBC cell lines were treated with β-catenin inhibitor iCRT14 at 5 µM for 48 h result in a significant reduction in the number of nucleoli per nucleus in TNBC cell lines. Representative photomicrographs of NucleolarID^TM^ stained cells are presented. The accompanying box plots present the average nucleoli/nucleus number for each cell line for the control and iCRT treated groups. Statistical analysis was performed using a *T*-test and error bars indicate SEM. **D** Graphical representation of Wnt signaling activity in HC11 cells throughout the process of differentiation. DIP = dexamethasone, insulin, prolactin cocktail for inducing differentiation. Total protein was isolated at 24, 48, and 72 h. Immunoblots for β-casein were used to verify the differentiation of HC11 cells. Immunoblots for active β-catenin (Phospho-Ser 552 is) and total β-catenin were performed to determine active β-catenin signaling. **E** Undifferentiated HC11 cells display higher numbers of nucleoli compared to differentiated HC11 cells based on AgNOR staining. Statistical significance was determined using a *T*-test and error bars represent SEM. **F** Treatment of HC11 cells with iCRT14 at 5 µM for 48 h resulted in a significant reduction in nucleolar number based on AgNOR staining. Statistical significance was determined using a *T*-test and error bars represent SEM.

Wnt/β-catenin signaling plays an integral role in TNBC progression. In normal-differentiated breast epithelium, β-catenin is present at the cell membrane as a part of catenin/cadherin junctions. In cancers, erroneous activation allows β-catenin to leave the membrane and translocate to the nucleus. There β-catenin acts as a transcription co-factor and drives transcription of proteins that support the epithelial-mesenchymal transition, invasion, and metastasis^31,32^. We stained breast tumor tissues, to evaluate the distribution of the β-catenin protein. As seen in Fig. 3A, TNBC specimens show remarkably high nuclear accumulation of β-catenin compared to non-TNBC specimens that show predominantly membranous staining. Next, we evaluated if there is a relationship between nucleolar numbers in TNBC cells and active β-catenin (defined as a ratio of nuclear/membranous β-catenin). We found that tumor tissues with active β-catenin signaling show an increased number of nucleoli per cell. More noticeably, TNBC specimens showed the concurrent presence of high nuclear β-catenin and high nucleolar number Fig. 3B.

Our observations in Fig. 2E show that about 75% of the significantly enriched nucleolar proteome of TNBCs (108 out of 145 proteins) comprises potential transcription targets of TCF/LEF. Our observations in Fig. 1 clearly show that TNBC cells have a greater number of nucleoli than non-TNBC cells. Therefore, we evaluated if inhibition of β-catenin would alter nucleolar numbers in TNBC cells. For this purpose, we used an inhibitor of catenin-related transcription (iCRT 14) that is highly specific to β-catenin driven transcription^33^. We conducted the experiment at a very low, sub-lethal dose (5 μM iCRT14). We observed that TNBC cell lines show a significant reduction in nucleolar number upon iCRT14 treatment (Fig. 3C). Studies using three additional, chemically dissimilar inhibitors of catenin-related transcription (ICG001, iCRT3, and LF3), at respective-sub-lethal doses, also showed a significant reduction in nucleolar number (Supplementary Fig. 2). This indicated that inhibition of β-catenin signaling significantly reduces the number of nucleoli.

It is well established that Wnt/β-catenin signaling is active in undifferentiated mammary epithelium and is involved in maintaining the bi-potent progenitor cell compartment^34^. However, upon differentiation of these cells, Wnt/β-catenin signaling ceases to be active^35^. In order to further understand if the process of differentiation impinges upon nucleolar numbers, we used HC11 (murine mammary epithelial cell line), an in vitro model of mammary differentiation^36^. Undifferentiated HC11 display remarkably high levels of β-catenin (Fig. 3D). Differentiation of HC11 is accompanied by increased levels of milk protein, β-casein. Using AgNOR staining, we observed that there is a significant reduction in nucleolar number in differentiated HC11 cells (Fig. 3E). Using this independent model system, we tested the effects of inhibiting β-catenin signaling on the nucleolar numbers in HC11. Our observations show that inhibition of β-catenin activity leads to a spectacular reduction in the nucleolar numbers in undifferentiated HC11 cells (Fig. 3F).

### Wnt inhibition causes changes in nucleolar content

**Fig. 4:**
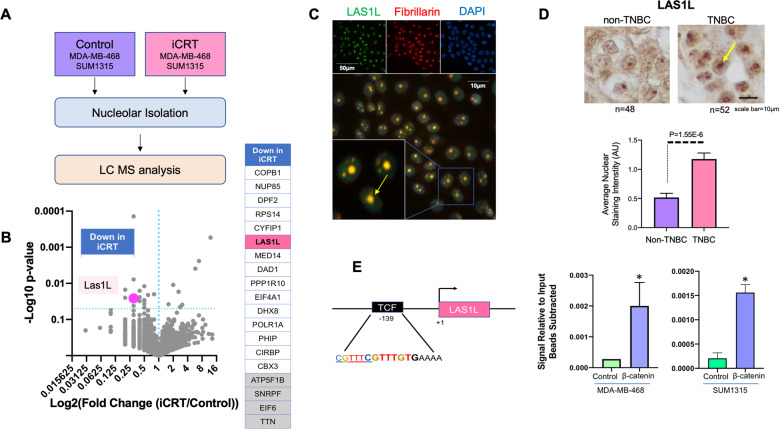
Nucleolar protein LAS1L is a target of β-catenin signaling. **A** Schematics of workflow for TNBC cells treated with 5 µM iCRT14 for 48 h for nucleolar isolation and proteomic analysis. **B** Nucleolar proteome of TNBC cells (SUM1315 and MDA-MB-468) was compared with and without iCRT14 treatment. Volcanoplot reveals 19 proteins (listed) significantly reduced in nucleoli of TNBC cells after iCRT14 treatment. Special attention is drawn to LAS1L, the only protein found to be significantly altered in both TNBC vs. non-TNBC proteomics as well as the iCRT14 nucleolar proteomics. **C** Immunofluorescent staining of LAS1L and Fibrillarin verifying the presence of LAS1L is a nucleolar protein in TNBC cells. **D** Immunohistochemical staining of TNBC vs. non-TNBC tumor samples for LAS1L verifies a significant increase in LAS1L staining in the TNBC tumor samples compared to Non-TNBC tumor samples. Statistical significance was determined by *T*-test and error bars to represent SEM. **E** Diagrammatic representation of the predicted TCF consensus sequence in the promoter region of the LAS1L gene. Is a tandem overlap of two TCR/LEF sites. CCTTTGAAC is the most preferred TCF/LEF binding cite^49^. Most conserved bases are indicated with red color. Bases less conserved are indicated with blue and yellow colors. ChIP-qPCR analysis of β-catenin bound to the TCF consensus sequence in the promoter of the LAS1L gene. Statistical significance was determined by *T*-test and error bars to represent SEM.

To gain further insight into changes in nucleolar content of TNBC cells after β-catenin inhibition, SUM1315 and MDA-MB 468 cells were treated with 5uM iCRT14 or vehicle control, and then nucleoli were isolated and evaluated using mass spectrometry analysis (schematic Fig. 4A). Using Scaffold^TM^ software, we determined that twenty-seven proteins changed significantly between the nucleoli of the two groups (Fig. 4B). Of these, nineteen proteins were significantly lower in the nucleoli of iCRT14 treated TNBCs. Using ENCODE ChIP-seq data, we found that fifteen out of these nineteen proteins could be potential targets of TCF/LEF signaling. Of these fifteen, LAS1L, stood out as a protein of significant interest for several reasons. LAS1L is a nucleolar endonuclease involved in pre-rRNA processing of internally transcribed sequence (ITS2) of 45S rDNA transcript. Thus, it directly influences the maturation of 28S rRNA, which is integral to the 60S ribosomal subunit. Therefore, LAS1L impacts the biogenesis of the 60S ribosomal subunit. LAS1L was also distinctively present as a significantly increased protein in the fifty “nucleolar confirmed” proteins in TNBC cells compared to non-TNBC cells. Our immunocytochemistry analysis confirms that LAS1L is indeed present in the nucleolus of TNBC cells (Fig. 4C). We then analyzed TNBC and non-TNBC tumor tissues for LAS1L protein levels using immunohistochemistry. We observed that indeed LAS1L expression is remarkably high in TNBC specimens. More specifically, the nucleolar staining in TNBC specimens tended to be more focused and defined (Fig. 4D). As an independent verification, we assessed publicly available RNAseq—IlluminaHiSeq for 1101 breast cancer primary tumors (cohort: TCGA Breast Cancer BRCA) using Xena browser^37^. The data were extracted for analysis of LAS1L mRNA expression and correlation with the PAM50 subtype. Indeed, we observed that TNBCs show significantly elevated mRNA expression of LAS1L (Supplementary Fig. 3A).

Based on ENCODE database, the LAS1L promoter has a predicted TCF/LEF site at position-139. Using ChIP-qPCR, we checked if this site is indeed recognized by β-catenin. Our results from both cell lines demonstrate that β-catenin clearly recognizes this region (Fig. 4E). Furthermore, we assessed the responsiveness of the predicted TCF consensus sequence in LAS1L by utilizing a luciferase reporter assay. We observed a significant reduction in luciferase activity after treatment with 5 µM iCRT (Supplementary Fig. 3B).

In addition, using immunoblotting, we detected a reduction of LAS1L levels in SUM1315 and MDA-MB-468 cells after treatment with iCRT14 (Supplementary Fig. 3C). Our query of gene expression omnibus (GEO) database^38^ revealed that in multiple myeloma cells, knockdown of β-catenin (Profile# GDS3578 / 208117_s_at) manifested as a significant reduction in LAS1L expression compared to control cells (Supplementary Fig. 3D). Based on these independent confirmations we conclude that LAS1L expression is transcriptionally regulated by β-catenin.

Our results (Fig. 3B) show that TNBC specimens with highly active (high nuclear/membrane ratio) β-catenin show elevated nucleolar numbers. Since LAS1L is a transcriptional target of β-catenin, we evaluated if breast cancer specimens with high LAS1L expression show an elevated number of nucleoli. Supplementary Fig. 3E shows the outcome of our analysis that revealed a striking relationship between LAS1L expression and increased nucleolar numbers.

### Knockdown of LAS1L leads to a reduction in metastatic potential

**Fig. 5:**
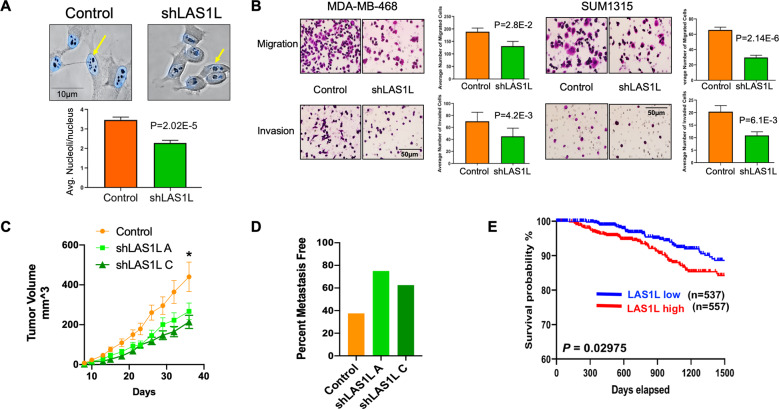
Knockdown of LAS1L leads to a reduction in tumorigenic and metastatic properties in TNBC. **A** Stable knockdown of LAS1L using short hairpin RNA results in a significant reduction in nucleolar number. Statistical significance was determined by *T*-test and error bars to represent SEM. **B** Knockdown of LAS1L in TNBC cells results in a significant reduction in the invasive and migratory capability of cells. Statistical significance was determined by *T*-test and error bars to represent SEM. **C** Growth of orthotopic xenografts of SUM1315 cells is significantly reduced following stable knockdown of LAS1L. Statistical significance was determined by two-way ANOVA followed by Dunnett’s post hoc test for tumor growth at respective time points and error bars represent SEM. *Represents significant statistical difference was reached for comparison of the control group compared to shLAS1 A as well as shLAS1L C growth curves for all days from day 18. **D** Lungs of mice with orthotopic xenografts of SUM1315 were examined for overt metastases. The incidence of metastasis was notably low for LAS1L knockout xenografts. **E** For survival analysis, the patient’s LAS1-L RNA expression data, measured by RNAseq—IlluminaHiSeq for 1101 breast cancer primary tumors (cohort: TCGA Breast Cancer BRCA) was accessed from a public data portal (https://xenabrowser.net) Sep. 2020. Data were extracted for analysis. Normalized RNA-seq data was used as LAS1-L gene expression values, and the median was used to classify samples into high and low expression groups for overall survival analysis. A Kaplan–Meier curve was generated, and a log-rank test applied.

To obtain more insight into the role of increased LAS1L in TNBC cells, we knocked-down LAS1L expression using short hairpin RNA. The resultant cells showed a significant reduction in the numbers of nucleoli per cell (Fig. 5A). We further analyzed various attributes of malignancy using the LAS1L silencing approach. Cells silenced for LAS1L showed decreased migration as measured by modified Boyden chamber assay. In addition, these cells showed highly compromised invasive ability in both, SUM1315 and MDA-MB 468 cell lines (Fig. 5B). We used SUM1315 cells stably silenced for LAS1L expression using two independent shRNAs to evaluate in vivo, the impact of LAS1L silencing. The resultant lines were partially silenced for LAS1L expression, the shLAS1L C line being silenced to a greater extent than shLAS1L A line (Supplementary Fig. 4A). Complete silencing of LAS1L may be lethal due to the key role played by LAS1L in ribosome biogenesis. This is because, consistent with published findings by Castle et al., knockdown of LAS1L in TNBC cells resulted in impaired biogenesis of large ribosomal subunit, which was observed as a decrease in the ratio of 60S to 40S subunit populations in sucrose gradient fractions, confirming Las1L’s key role in the biogenesis of 60S ribosomal subunit (Supplementary Fig. 4B)^39–41^. We evaluated orthotopic (murine mammary fat pad) xenograft growth of SUM1315 cells stably silenced for LAS1L. LAS1L-silenced cells showed comparable tumor take rates; however, tumor growth was significantly reduced (Fig. 5C). We analyzed these xenografts for LAS1L expression levels using immunohistochemistry. We observed that the xenografts indeed maintained reduced LAS1L expression (Supplementary Fig. 4C). We used AgNOR staining to analyze these xenografts for nucleolar numbers. As seen in Supplementary Fig. 4D both, shLAS1L A and C silenced tumor tissues showed significantly fewer nucleolar numbers compared to the control. More strikingly, we observed a reduced incidence of lung metastasis in these mice (Fig. 5D). To understand if elevated LAS1L expression in breast cancer provides insight into prognosis, we assessed publicly available RNAseq—IlluminaHiSeq for 1101 breast cancer primary tumors (cohort: TCGA Breast Cancer BRCA) using Xena browser^37^. Normalized RNA-seq data was used as LAS1L gene expression values, and the median was used to classify samples into high and low expression groups for overall survival analysis. A Kaplan–Meier curve was generated and a log-rank test applied. As seen in Fig. 5E, breast cancer patients with higher LAS1L expression levels show significantly poor prognosis (*P* = 0.029).

## Discussion

Classical pathologic diagnosis of tumor tissue has revealed that nucleolar hypertrophy and increased nucleolar number present as predictive and prognostic parameters of increased mortality^4,6,42^. Newly emerging evidence indicates that quantitative and qualitative changes in ribosomes are vitally involved in metastasis^7,20,43^. The dysregulated nucleolar morphology and increased numbers reflect hyperactivation of rDNA transcription consistent with increased ribosome biogenesis that correlates with adverse prognosis^4,6^. With the adoption of AgNOR staining as a reliable marker for tumorigenic growth and a prognostic indicator for cancer patient response to therapy and survival, the nucleolus has successfully bridged clinical and basic science^4^. Interestingly, despite its recognized value as a prognostic indicator, relatively little is known about the functional contributions of the nucleolus in cancer and in particular, in metastasis.

Our observations from a cohort of TNBC and non-TNBC specimens revealed that TNBCs show significantly increased numbers of nucleoli per cell, compared to non-TNBC specimens. The proteome of the nucleolus is highly dynamic, and it reflects the pathophysiologic state of the cell and its translational needs. Thus, we searched for clues to the differences in TNBCs by comparing their nucleolar proteome to non-TNBC’s. We noticed that the differences in the proteomes were driven by an ensemble of proteins that regulated ribosome biogenesis. The unexpected finding that emerged through this analysis was that 75% of the differentially enriched nucleolar proteome of TNBC cells encompassed proteins that were potential transcription targets of β-catenin signaling. Wnt/β-catenin signaling plays a crucial role in cell fate determination, cell polarity, and cell proliferation during embryonic development. This pathway is also critical in the development of mammary ductal epithelium; specifically, in the maintenance of the mammary ductal progenitor cell population, and it influences luminal differentiation^44^. This pathway is dysregulated in multiple malignancies including the breast and is distinctly active in TNBCs. Moreover, β-catenin activation is noticeably enriched in basal-like breast cancers and predicts poor outcome^25^.

We observed that inhibition of β-catenin led to reduced nucleolar numbers in TNBC cell lines. Our observations in the HC11 model of mammary epithelial differentiation offer independent verification of this relationship of reduced nucleolar numbers following inhibition of β-catenin. The Wnt signaling pathway is a group of multiple signaling streams such as canonical (Wnt/ β-catenin) and non-canonical, as well as Wnt-STOP signaling. Multiple studies using different model systems have provided mechanistic insights into the regulation of ribosome biogenesis via these various streams^45–48^. Our studies specifically unraveled the contributions of β-catenin signaling in TNBC nucleolar biology.

Using nucleolar proteomic analysis of β-catenin inhibited TNBC cells we uncovered an interesting insight into the regulation of ribosome biogenesis by β-catenin. Ribosome biogenesis is one of the most energy-demanding processes, involving a large number of assembly and maturation factors, the functions of which are orchestrated by multiple cellular inputs, including mitogenic signals. There is a suggestion of a broader role for dysregulated ribosome biogenesis in the development and progression of cancers^42^. The mature 80S ribosome comprises a 40S subunit with a single 18S rRNA and a 60S subunit-containing 5S, 5.8S, and 28S rRNAs. LAS1L stood out as an important player in β-catenin-regulated TNBC nucleolar functions as it is involved in the processing of the 47 S pre-rRNA to 28S and 5.8S rRNAs and is required for the synthesis of the 60S ribosomal subunit^40^. Detailed elucidation of the activities of the LAS1L containing Rix1 complex in regulating ribosome biogenesis in yeast and mammalian systems are already available^39,41^. Our study highlights the requirement of LAS1L expression for the aggressive phenotypes of TNBCs. We observed that silencing LAS1L impacted cell proliferation (Supplementary Fig. 4E). This is not surprising as it is an obvious consequence of impaired ribosome biogenesis. However, silencing LAS1L also compromised the migratory and invasive abilities of TNBC cells. Most noticeable was the reduced in vivo tumor growth rates following LAS1L silencing and the impaired ability to metastasize that was reflected in reduced incidence of metastasis to lungs. This could be because the LAS1L silenced cells are less adept at facing the challenges posed by the complex in vivo microenvironment, due to compromised ribosome biogenesis, which limits the translational adaptability needed for survival. Conceivably, a total knockdown of LAS1L may not be viable. The extent of silencing achieved by the shRNA was comparable to the reduced levels by the low concentration of iCRT14, and this possibly allowed us to document the biological impacts. It is important to note that we observed that LAS1L expression is elevated in TNBC specimens and its silencing blunted the attributes of malignancy of TNBC cells. It is noticeable that TCGA datasets reveal that patients with increased LAS1L expression show poor prognoses. Thus, overall our observations highlight the dependence of TNBC on elevated ribosome biogenesis demands and highlight the importance of understanding the contributions of rRNA metabolism and ribosome biogenesis process in tumor progression.

## Supplementary information

Supplementary Figure Legends

Supplementary Figure 1

Supplementary Figure 2

Supplementary Figure 3

Supplementary Figure 4

Table 1

Table 2
