# Supplementary material for: Inhibiting β-catenin disables nucleolar functions in triple-negative breast cancer
Source: Cell Death Dis. 2021 Mar 4;12(3):242. doi: 10.1038/s41419-021-03531-z (PMC7933177; doi:10.1038/s41419-021-03531-z)
Supplement: Supplementary file 1 — Supplementary Figure Legends [file 41419_2021_3531_MOESM1_ESM.docx]

**Supplementary Figure Legends:**

**Supplementary Figure 1:** Heat map illustrating the 100 most significantly enriched proteins in TNBC cell nucleoli, compared to the 100 most significantly enriched proteins in the Non TNBC cell nucleoli.

**Supplementary Figure 2:** Inhibition of β-catenin signaling with multiple different inhibitors of β-catenin transcription results in a significant reduction in nucleolar number in SUM1315 cells. Statistical significance was determined by T-Test and error bars represent SEM.

**Supplementary Figure 3:**

1. Patient’s LAS1L RNA expression data measured by RNAseq - IlluminaHiSeq for 1101 breast cancer primary tumors (cohort: TCGA Breast Cancer BRCA) was accessed from public data portal (https://xenabrowser.net)^37^. Data were extracted for analysis and LAS1L RNA expression were examined in correlation of PAM50 subtype (PAM50-Call RNA-seq) One-way Anova method was used for statistical analysis using GraphPad Prism version 8. Comparisons were considered statistically significant for p-value < 0.05.
2. Luciferase reporter assay specific to TCF consensus sequence in the LAS1L promoter region confirms that LAS1L is a target of β-catenin signaling.
3. Immunoblotting confirms proteomics findings that treatment of TNBC cells with iCRT14 results in the reduction of LAS1L protein.
4. GeoData studies in multiple myeloma cells depleted for beta catenin results in a corresponding reduction in LAS1L expression.
5. Immunohistochemical staining indicated a correlation with increased LAS1L and increased nucleolar number found in TNBC tumor samples compared to non-TNBC tumor samples.

**Supplementary Figure 4:**

1. Immunoblotting verification of reduction on LAS1L protein levels after stable transduction with short hairpin RNA against LAS1L.
2. Stable knock down of LAS1L in SUM1315 cells results in a reduction in the ration of 60S to 40S ribosomal subunits confirming LAS1L’s previously established role in ribosome biogenesis.
3. Immunohistochemical staining of tumor xenografts from SUM1315 cells confirming knock down for LAS1L protein in tumors.
4. Tumor xenografts formed from SUM1315 cells stabily knocked down for LAS1L exhibit a significant reduction in the number of nucleoli per nucleus when compared to tumors formed from control tumors.
5. Stable knockdown of LAS1L in SUM1315 cells results in a significant reduction in proliferation after 72 hours. Statistical significance was determined by T-Test and error bars represent SEM.
