# Supplementary figures and images for: Inhibiting β-catenin disables nucleolar functions in triple-negative breast cancer
Source: Cell Death Dis. 2021 Mar 4;12(3):242. doi: 10.1038/s41419-021-03531-z (PMC7933177; doi:10.1038/s41419-021-03531-z)

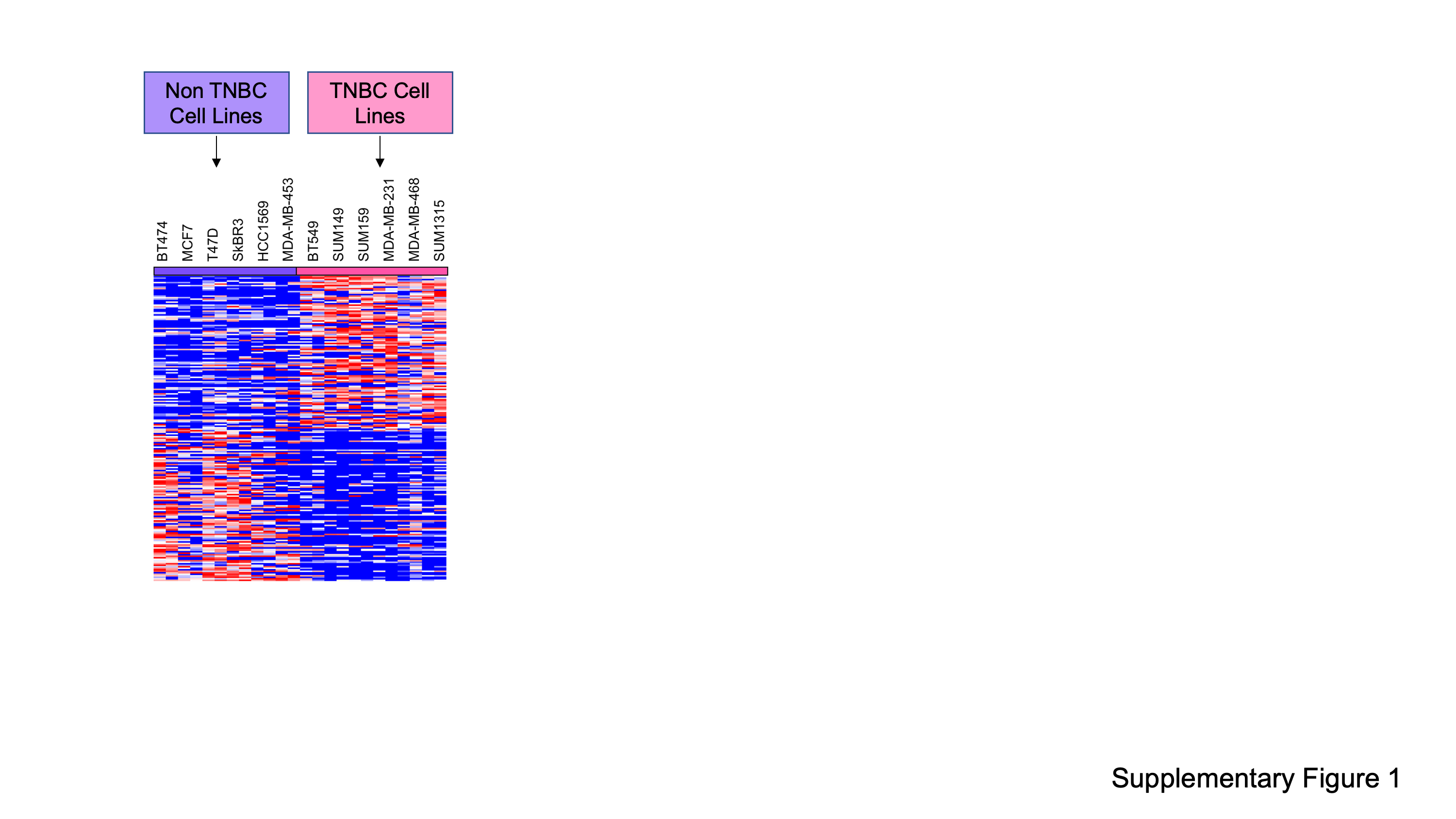

Supplement: Supplementary file 2 — Supplementary Figure 1 [file 41419_2021_3531_MOESM2_ESM.tif]

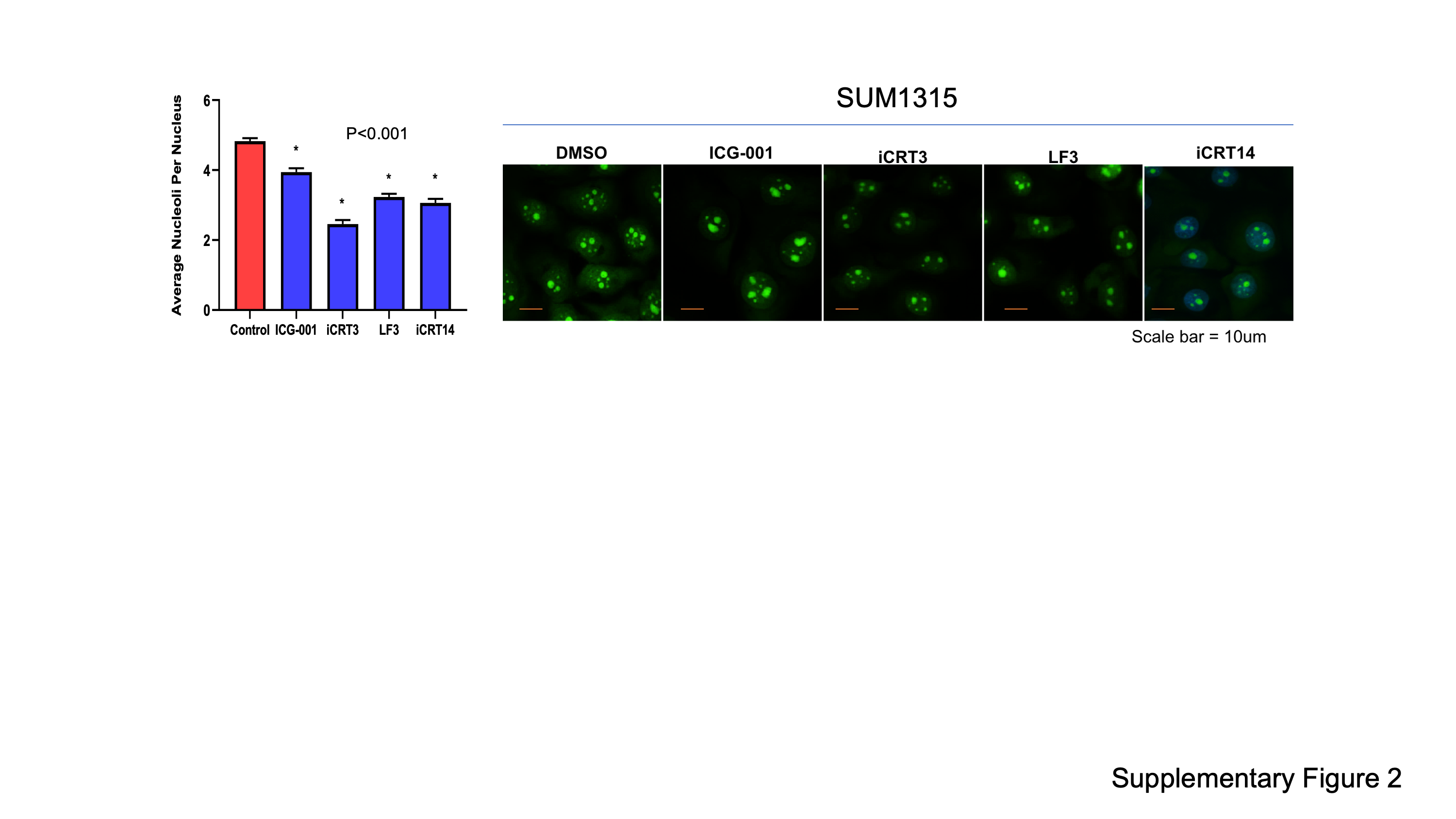

Supplement: Supplementary file 3 — Supplementary Figure 2 [file 41419_2021_3531_MOESM3_ESM.tif]

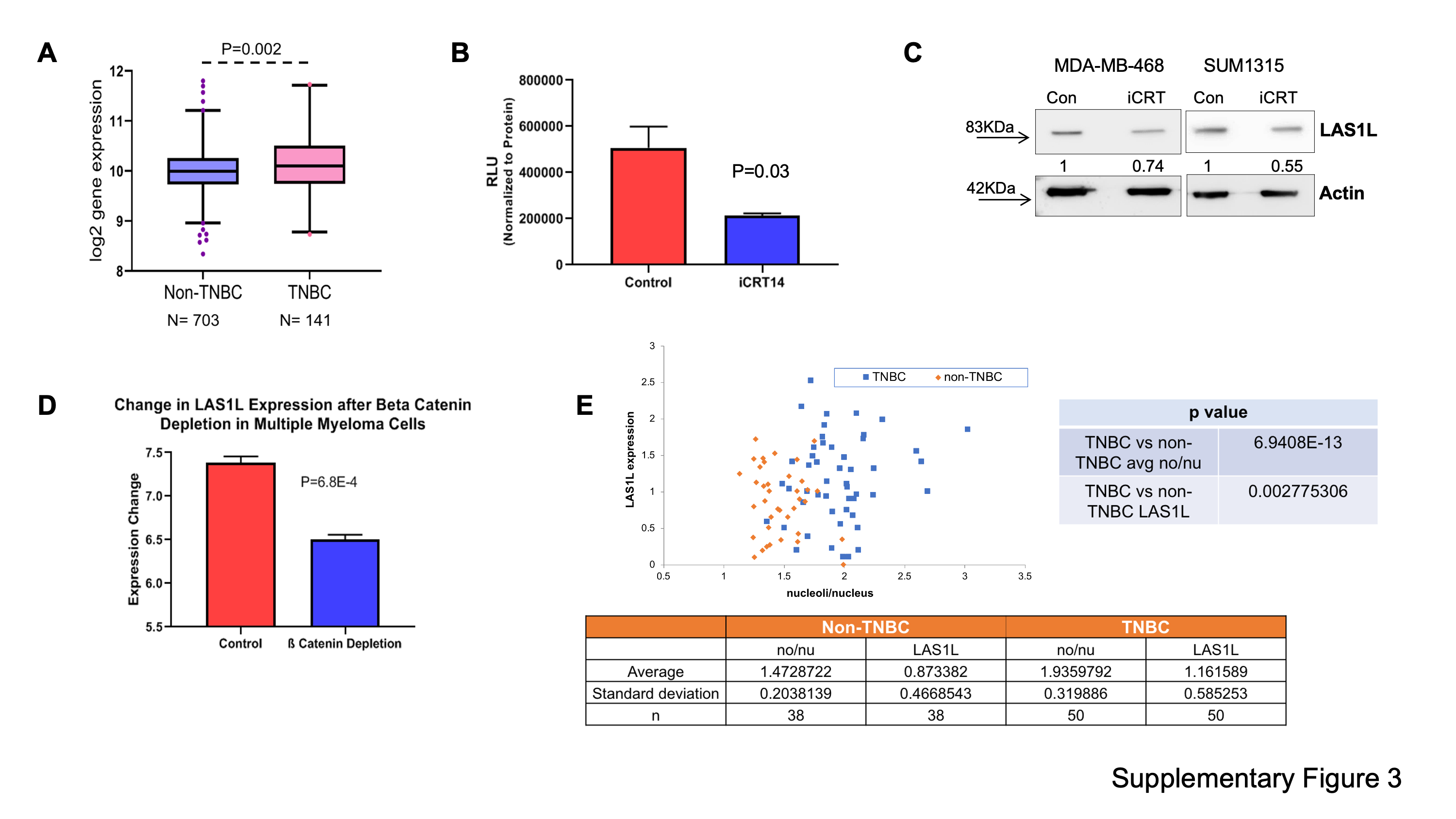

Supplement: Supplementary file 4 — Supplementary Figure 3 [file 41419_2021_3531_MOESM4_ESM.tif]

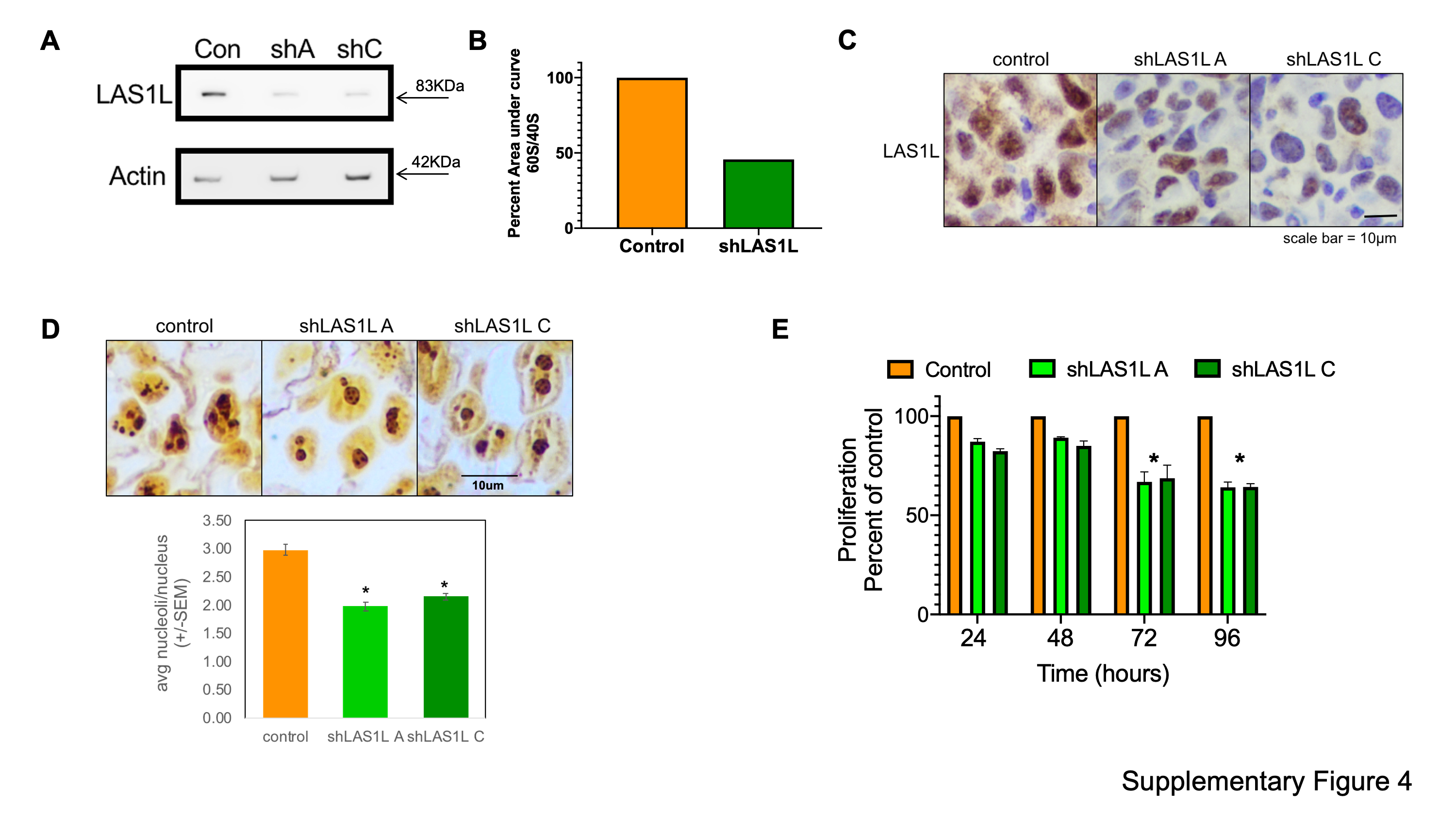

Supplement: Supplementary file 5 — Supplementary Figure 4 [file 41419_2021_3531_MOESM5_ESM.tif]
