# Supplementary material for: Inhibiting β-catenin disables nucleolar functions in triple-negative breast cancer
Source: Cell Death Dis. 2021 Mar 4;12(3):242. doi: 10.1038/s41419-021-03531-z (PMC7933177; doi:10.1038/s41419-021-03531-z)
Supplement: Supplementary file 6 — Table 1 [file 41419_2021_3531_MOESM6_ESM.docx]

| **Table 1** |  |  |  |
| --- | --- | --- | --- |
|  | **Proteins enriched in nucleoli of TNBC cells** |  |  |
|  | **Identified Proteins** | **Protein Accession Number** | **Gene name** |
| **1** | Protein AATF n=1 Tax=Homo sapiens RepID=AATF_HUMAN | Q9NY61 | AATF |
| **2** | ATP-binding cassette sub-family E member 1 n=9 Tax=Euarchontoglires RepID=ABCE1_HUMAN | P61221 | ABCE1 |
| **3** | Activator of basal transcription 1 n=5 Tax=Homininae RepID=ABT1_HUMAN | Q9ULW3 | ABT1 |
| **4** | Alpha-actinin-1 n=5 Tax=Hominoidea RepID=ACTN1_HUMAN | P12814 | ACTN1 |
| **5** | Apoptosis inhibitor 5 n=6 Tax=Simiiformes RepID=API5_HUMAN | Q9BZZ5 | API5 |
| **6** | AT-rich interactive domain-containing protein 1B n=1 Tax=Homo sapiens RepID=ARI1B_HUMAN | Q8NFD5 | ARID1B |
| **7** | Caveolin-1 n=10 Tax=Catarrhini RepID=CAV1_HUMAN | Q03135 | CAV1 |
| **8** | G2/mitotic-specific cyclin-B1 n=1 Tax=Homo sapiens RepID=CCNB1_HUMAN | P14635 | CCNB1 |
| **9** | T-complex protein 1 subunit epsilon n=12 Tax=Catarrhini RepID=TCPE_HUMAN | P48643 | CCT5 |
| **10** | T-complex protein 1 subunit eta n=3 Tax=Homininae RepID=TCPH_HUMAN | Q99832 | CCT7 |
| **11** | Coronin-1C n=7 Tax=Homo sapiens RepID=COR1C_HUMAN | Q9ULV4 | CORO1C |
| **12** | DDB1- and CUL4-associated factor 13 n=3 Tax=Homo sapiens RepID=DCA13_HUMAN | Q9NV06 | DCAF13 |
| **13** | ATP-dependent RNA helicase DDX39A n=13 Tax=Catarrhini RepID=DX39A_HUMAN | O00148 | DDX39A |
| **14** | Probable ATP-dependent RNA helicase DDX47 n=3 Tax=Homo sapiens RepID=DDX47_HUMAN | Q9H0S4 | DDX47 |
| **15** | Probable ATP-dependent RNA helicase DDX49 n=2 Tax=Homininae RepID=DDX49_HUMAN | Q9Y6V7 | DDX49 |
| **16** | ATP-dependent RNA helicase DDX50 n=6 Tax=Catarrhini RepID=DDX50_HUMAN | Q9BQ39 | DDX50 |
| **17** | Probable ATP-dependent RNA helicase DDX56 n=3 Tax=Homininae RepID=DDX56_HUMAN | Q9NY93 | DDX56 |
| **18** | H/ACA ribonucleoprotein complex subunit 4 n=3 Tax=Homininae RepID=DKC1_HUMAN | O60832 | DKC1 |
| **19** | DNA methyltransferase 1-associated protein 1 n=9 Tax=Simiiformes RepID=DMAP1_HUMAN | Q9NPF5 | DMAP1 |
| **20** | DnaJ homolog subfamily C member 10 n=2 Tax=Homo sapiens RepID=DJC10_HUMAN | Q8IXB1 | DNAJC10 |
| **21** | Deoxynucleotidyltransferase terminal-interacting protein 2 n=1 Tax=Homo sapiens RepID=TDIF2_HUMAN | Q5QJE6 | DNTTIP2 |
| **22** | Cytoplasmic dynein 1 heavy chain 1 n=4 Tax=Hominoidea RepID=DYHC1_HUMAN | Q14204 | DYNC1H1 |
| **23** | Probable rRNA-processing protein EBP2 n=3 Tax=Homo sapiens RepID=EBP2_HUMAN | Q99848 | EBNA1BP2 |
| **24** | EH domain-containing protein 2 n=6 Tax=Homininae RepID=EHD2_HUMAN | Q9NZN4 | EHD2 |
| **25** | ER membrane protein complex subunit 8 n=14 Tax=Simiiformes RepID=EMC8_HUMAN | O43402 | EMC8 |
| **26** | rRNA 2'-O-methyltransferase fibrillarin n=3 Tax=Homininae RepID=FBRL_HUMAN | P22087 | FBL |
| **27** | Flotillin-2 n=7 Tax=Catarrhini RepID=FLOT2_HUMAN | Q14254 | FLOT2 |
| **28** | Transcriptional repressor p66-alpha n=2 Tax=Homo sapiens RepID=P66A_HUMAN | Q86YP4 | GATAD2A |
| **29** | Growth hormone-inducible transmembrane protein n=2 Tax=Homo sapiens RepID=GHITM_HUMAN | Q9H3K2 | GHITM |
| **30** | Guanine nucleotide-binding protein G(k) subunit alpha n=9 Tax=Catarrhini RepID=GNAI3_HUMAN | P08754 | GNAI3 |
| **31** | Guanine nucleotide-binding protein-like 3 n=3 Tax=Homo sapiens RepID=GNL3_HUMAN | Q9BVP2 | GNL3 |
| **32** | Nucleolar GTP-binding protein 1 n=4 Tax=Homo sapiens RepID=NOG1_HUMAN | Q9BZE4 | GTPBP4 |
| **33** | High mobility group protein HMG-I/HMG-Y n=9 Tax=Boreoeutheria RepID=HMGA1_HUMAN | P17096 | HMGA1 |
| **34** | High mobility group nucleosome-binding domain-containing protein 4 n=6 Tax=Catarrhini RepID=HMGN4_HUMAN | O00479 | HMGN4 |
| **35** | Heterogeneous nuclear ribonucleoprotein R n=10 Tax=Boreoeutheria RepID=HNRPR_HUMAN | O43390 | HNRNPR |
| **36** | Transcription factor AP-1 n=2 Tax=Homininae RepID=JUN_HUMAN | P05412 | JUN |
| **37** | Kinesin-like protein KIF20A n=3 Tax=Homo sapiens RepID=KI20A_HUMAN | O95235 | KIF20A |
| **38** | Kinesin-like protein KIF2C n=3 Tax=Homo sapiens RepID=KIF2C_HUMAN | Q99661 | KIF2C |
| **39** | Kinesin-1 heavy chain n=2 Tax=Homo sapiens RepID=KINH_HUMAN | P33176 | KIF5B |
| **40** | Ribosomal biogenesis protein LAS1L n=2 Tax=Homo sapiens RepID=LAS1L_HUMAN | Q9Y4W2 | LAS1L |
| **41** | Microtubule-associated protein 4 n=2 Tax=Homo sapiens RepID=MAP4_HUMAN | P27816 | MAP4 |
| **42** | Protein max n=13 Tax=Eutheria RepID=MAX_HUMAN | P61244 | MAX |
| **43** | Midasin n=4 Tax=Homo sapiens RepID=MDN1_HUMAN | Q9NU22 | MDN1 |
| **44** | Methionine aminopeptidase 1 n=1 Tax=Homo sapiens RepID=MAP11_HUMAN | P53582 | METAP1 |
| **45** | Antigen KI-67 n=2 Tax=Homo sapiens RepID=KI67_HUMAN | P46013 | MKI67 |
| **46** | Myosin phosphatase Rho-interacting protein n=1 Tax=Homo sapiens RepID=MPRIP_HUMAN | Q6WCQ1 | MPRIP |
| **47** | Myb-binding protein 1A n=1 Tax=Homo sapiens RepID=MBB1A_HUMAN | Q9BQG0 | MYBBP1A |
| **48** | Myosin regulatory light chain 12A n=8 Tax=Hominidae RepID=ML12A_HUMAN | P19105 | MYL12A |
| **49** | N-acetyltransferase 10 n=2 Tax=Homo sapiens RepID=NAT10_HUMAN | Q9H0A0 | NAT10 |
| **50** | Nuclear cap-binding protein subunit 1 n=6 Tax=Catarrhini RepID=NCBP1_HUMAN | Q09161 | NCBP1 |
| **51** | Nucleolar complex protein 2 homolog n=1 Tax=Homo sapiens RepID=NOC2L_HUMAN | Q9Y3T9 | NOC2L |
| **52** | Nucleolar complex protein 4 homolog n=1 Tax=Homo sapiens RepID=NOC4L_HUMAN | Q9BVI4 | NOC4L |
| **53** | Nucleolar protein 11 n=2 Tax=Homo sapiens RepID=NOL11_HUMAN | Q9H8H0 | NOL11 |
| **54** | Nucleolar protein 16 n=3 Tax=Homininae RepID=NOP16_HUMAN | Q9Y3C1 | NOP16 |
| **55** | Probable 28S rRNA (cytosine(4447)-C(5))-methyltransferase n=1 Tax=Homo sapiens RepID=NOP2_HUMAN | P46087 | NOP2 |
| **56** | Nuclear pore complex protein Nup160 n=2 Tax=Homo sapiens RepID=NU160_HUMAN | Q12769 | NUP160 |
| **57** | PHD finger protein 3 n=9 Tax=Homo sapiens RepID=PHF3_HUMAN | Q92576 | PHF3 |
| **58** | Peptidyl-prolyl cis-trans isomerase H n=30 Tax=Eutheria RepID=PPIH_HUMAN | O43447 | PPIH |
| **59** | Peroxiredoxin-1 n=3 Tax=Homininae RepID=PRDX1_HUMAN | Q06830 | PRDX1 |
| **60** | Peroxiredoxin-2 n=6 Tax=Catarrhini RepID=PRDX2_HUMAN | P32119 | PRDX2 |
| **61** | Protein arginine N-methyltransferase 1 n=11 Tax=Boreoeutheria RepID=ANM1_HUMAN | Q99873 | PRMT1 |
| **62** | Pre-mRNA-splicing factor 38A n=23 Tax=Eutheria RepID=PR38A_HUMAN | Q8NAV1 | PRPF38A |
| **63** | 60 kDa U4/U6 snRNP-specific spliceosomal protein n=4 Tax=Homininae RepID=Q5T1M7_HUMAN | Q5T1M7 (+1) | PRPF4 |
| **64** | PC4 and SFRS1-interacting protein n=4 Tax=Homininae RepID=PSIP1_HUMAN | O75475 | PSIP1 |
| **65** | Proteasome subunit alpha type-5 n=40 Tax=Eutheria RepID=PSA5_HUMAN | P28066 | PSMA5 |
| **66** | 26S protease regulatory subunit 8 n=20 Tax=Boreoeutheria RepID=PRS8_HUMAN | P62195 | PSMC5 |
| **67** | Polypyrimidine tract-binding protein 3 n=3 Tax=Homininae RepID=PTBP3_HUMAN | O95758 | PTBP3 |
| **68** | Protein quaking n=12 Tax=Boreoeutheria RepID=QKI_HUMAN | Q96PU8 | QKI |
| **69** | RNA binding motif protein, X-linked-like-1 n=2 Tax=Homo sapiens RepID=RMXL1_HUMAN | Q96E39 | RBMXL1 |
| **70** | RNA exonuclease 4 n=3 Tax=Homo sapiens RepID=REXO4_HUMAN | Q9GZR2 | REXO4 |
| **71** | 60S ribosomal protein L13a n=6 Tax=Boreoeutheria RepID=RL13A_HUMAN | P40429 | RPL13A |
| **72** | RPL14 protein n=1 Tax=Homo sapiens RepID=Q6IPH7_HUMAN | Q6IPH7 | RPL14 |
| **73** | 60S ribosomal protein L23 n=45 Tax=Euteleostomi RepID=RL23_HUMAN | P62829 | RPL23 |
| **74** | 60S ribosomal protein L29 n=1 Tax=Homo sapiens RepID=RL29_HUMAN | P47914 (+1) | RPL29 |
| **75** | 60S ribosomal protein L31 n=32 Tax=Amniota RepID=RL31_HUMAN | P62899 | RPL31 |
| **76** | 60S ribosomal protein L36 n=16 Tax=Boreoeutheria RepID=RL36_HUMAN | Q9Y3U8 | RPL36 |
| **77** | 60S ribosomal protein L4 n=2 Tax=Homo sapiens RepID=RL4_HUMAN | P36578 | RPL4 |
| **78** | 40S ribosomal protein S11 n=28 Tax=Eutheria RepID=RS11_HUMAN | P62280 | RPS11 |
| **79** | 40S ribosomal protein S20 n=20 Tax=Boreoeutheria RepID=RS20_HUMAN | P60866 | RPS20 |
| **80** | 40S ribosomal protein S25 n=21 Tax=Amniota RepID=RS25_HUMAN | P62851 | RPS25 |
| **81** | 40S ribosomal protein S5 n=22 Tax=Theria RepID=RS5_HUMAN | P46782 | RPS5 |
| **82** | 40S ribosomal protein SA n=4 Tax=Hominoidea RepID=RSSA_HUMAN | P08865 | RPSA |
| **83** | Ribosomal RNA processing protein 1 homolog A n=1 Tax=Homo sapiens RepID=RRP1_HUMAN | P56182 | RRP1 |
| **84** | RRP15-like protein n=1 Tax=Homo sapiens RepID=RRP15_HUMAN | Q9Y3B9 | RRP15 |
| **85** | Ribosomal RNA processing protein 1 homolog B n=1 Tax=Homo sapiens RepID=RRP1B_HUMAN | Q14684 | RRP1B |
| **86** | RuvB-like 1 n=17 Tax=Boreoeutheria RepID=RUVB1_HUMAN | Q9Y265 | RUVBL1 |
| **87** | RuvB-like 2 n=8 Tax=Simiiformes RepID=RUVB2_HUMAN | Q9Y230 | RUVBL2 |
| **88** | Protein S100-A4 n=8 Tax=Catarrhini RepID=S10A4_HUMAN | P26447 | S100A4 |
| **89** | SAP30-binding protein n=6 Tax=Hominoidea RepID=S30BP_HUMAN | Q9UHR5 | SAP30BP |
| **90** | Protein transport protein Sec61 subunit beta n=18 Tax=Boreoeutheria RepID=SC61B_HUMAN | P60468 | SEC61B |
| **91** | U1 small nuclear ribonucleoprotein 70 kDa n=8 Tax=Catarrhini RepID=RU17_HUMAN | P08621 | SNRNP70 |
| **92** | Small nuclear ribonucleoprotein Sm D2 n=21 Tax=Euteleostomi RepID=SMD2_HUMAN | P62316 | SNRPD2 |
| **93** | Small nuclear ribonucleoprotein E n=24 Tax=Amniota RepID=RUXE_HUMAN | P62304 | SNRPE |
| **94** | Signal peptidase complex subunit 2 n=1 Tax=Homo sapiens RepID=SPCS2_HUMAN | Q15005 | SPCS2 |
| **95** | Heterogeneous nuclear ribonucleoprotein Q n=14 Tax=Boreoeutheria RepID=HNRPQ_HUMAN | O60506 | SYNCRIP |
| **96** | Transgelin-2 n=8 Tax=Catarrhini RepID=TAGL2_HUMAN | P37802 | TAGLN2 |
| **97** | Transducin beta-like protein 3 n=2 Tax=Homo sapiens RepID=TBL3_HUMAN | Q12788 | TBL3 |
| **98** | Transforming growth factor-beta-induced protein ig-h3 n=3 Tax=Homininae RepID=BGH3_HUMAN | Q15582 | TGFBI |
| **99** | THO complex subunit 6 homolog n=2 Tax=Homo sapiens RepID=THOC6_HUMAN | Q86W42 | THOC6 |
| **100** | Transmembrane protein 43 n=2 Tax=Homo sapiens RepID=TMM43_HUMAN | Q9BTV4 | TMEM43 |
| **101** | Targeting protein for Xklp2 n=2 Tax=Homo sapiens RepID=TPX2_HUMAN | Q9ULW0 | TPX2 |
| **102** | Transformer-2 protein homolog alpha n=12 Tax=Euarchontoglires RepID=TRA2A_HUMAN | Q13595 | TRA2A |
| **103** | U2 snRNP-associated SURP motif-containing protein n=3 Tax=Boreoeutheria RepID=SR140_HUMAN | O15042 | U2SURP |
| **104** | U3 small nucleolar RNA-associated protein 14 homolog A n=2 Tax=Homo sapiens RepID=UT14A_HUMAN | Q9BVJ6 | UTP14A |
| **105** | Synaptic vesicle membrane protein VAT-1 homolog n=7 Tax=Homo sapiens RepID=VAT1_HUMAN | Q99536 | VAT1 |
| **106** | Vimentin n=4 Tax=Homininae RepID=VIME_HUMAN | P08670 | VIM |
| **107** | WD repeat-containing protein 18 n=1 Tax=Homo sapiens RepID=WDR18_HUMAN | Q9BV38 | WDR18 |
| **108** | WD repeat-containing protein 5 n=26 Tax=Boreoeutheria RepID=WDR5_HUMAN | P61964 | WDR5 |
| **109** | Protein DEK n=2 Tax=Catarrhini RepID=DEK_HUMAN | P35659 | DEK |
| **110** | 60S ribosomal protein L3 n=5 Tax=Catarrhini RepID=RL3_HUMAN | P39023 | RPL3 |
| **111** | Cell cycle and apoptosis regulator protein 2 n=4 Tax=Homo sapiens RepID=CCAR2_HUMAN | Q8N163 | CCAR2 |
| **112** | NHP2-like protein 1 n=27 Tax=Eutheria RepID=NH2L1_HUMAN | P55769 | SNU13 |
| **113** | Pinin n=3 Tax=Homo sapiens RepID=PININ_HUMAN | Q9H307 | PNN |
| **114** | 60S ribosomal protein L6 n=7 Tax=Homininae RepID=RL6_HUMAN | Q02878 | RPL6 |
| **115** | 40S ribosomal protein S8 n=30 Tax=Boreoeutheria RepID=RS8_HUMAN | P62241 | RPS8 |
| **116** | WD repeat-containing protein 36 n=2 Tax=Homo sapiens RepID=WDR36_HUMAN | Q8NI36 | WDR36 |
| **117** | CCAAT/enhancer-binding protein zeta n=1 Tax=Homo sapiens RepID=CEBPZ_HUMAN | Q03701 | CEBPZ |
| **118** | MKI67 FHA domain-interacting nucleolar phosphoprotein n=2 Tax=Homo sapiens RepID=MK67I_HUMAN | Q9BYG3 | NIFK |
| **119** | Myosin light polypeptide 6 n=12 Tax=Boreoeutheria RepID=MYL6_HUMAN | P60660 | MYL6 |
| **120** | Splicing factor 3B subunit 6 n=55 Tax=Euteleostomi RepID=SF3B6_HUMAN | Q9Y3B4 | SF3B6 |
| **121** | Protein AHNAK2 n=3 Tax=Homo sapiens RepID=AHNK2_HUMAN | Q8IVF2 | AHNAK2 |
| **122** | MAX gene-associated protein n=1 Tax=Homo sapiens RepID=MGAP_HUMAN | Q8IWI9 | MGA |
| **123** | Polymerase I and transcript release factor n=1 Tax=Homo sapiens RepID=PTRF_HUMAN | Q6NZI2 | CAVIN1 |
| **124** | DNA-directed RNA polymerases I, II, and III subunit RPABC1 n=2 Tax=Homo sapiens RepID=RPAB1_HUMAN | P19388 | POLR2E |
| **125** | Ribosomal RNA small subunit methyltransferase NEP1 n=2 Tax=Homininae RepID=NEP1_HUMAN | Q92979 | EMG1 |
| **126** | U5 small nuclear ribonucleoprotein 40 kDa protein n=9 Tax=Catarrhini RepID=SNR40_HUMAN | Q96DI7 | SNRNP40 |
| **127** | T-complex protein 1 subunit theta n=4 Tax=Hominidae RepID=TCPQ_HUMAN | P50990 | CCT8 |
| **128** | U6 snRNA-associated Sm-like protein LSm6 n=65 Tax=Sarcopterygii RepID=LSM6_HUMAN | P62312 | LSM6 |
| **129** | Annexin A1 n=4 Tax=Homininae RepID=ANXA1_HUMAN | P04083 | ANXA1 |
| **130** | U3 small nucleolar RNA-interacting protein 2 n=1 Tax=Homo sapiens RepID=U3IP2_HUMAN | O43818 | RRP9 |
| **131** | Neuroguidin n=3 Tax=Homo sapiens RepID=NGDN_HUMAN | Q8NEJ9 | NGDN |
| **132** | Histone-lysine N-methyltransferase NSD2 n=2 Tax=Homo sapiens RepID=NSD2_HUMAN | O96028 | NSD2 |
| **133** | Unhealthy ribosome biogenesis protein 2 homolog n=2 Tax=Homo sapiens RepID=URB2_HUMAN | Q14146 | URB2 |
| **134** | Bystin n=2 Tax=Homininae RepID=BYST_HUMAN | Q13895 | BYSL |
| **135** | WD repeat-containing protein 74 n=2 Tax=Homo sapiens RepID=WDR74_HUMAN | Q6RFH5 | WDR74 |
| **136** | Serum response factor-binding protein 1 n=1 Tax=Homo sapiens RepID=SRFB1_HUMAN | Q8NEF9 | SRFBP1 |
| **137** | Putative ATP-dependent RNA helicase DHX33 n=2 Tax=Homo sapiens RepID=DHX33_HUMAN | Q9H6R0 | DHX33 |
| **138** | Ephrin type-A receptor 2 n=3 Tax=Homo sapiens RepID=EPHA2_HUMAN | P29317 | EPHA2 |
| **139** | Filamin-C n=2 Tax=Homo sapiens RepID=FLNC_HUMAN | Q14315 | FLNC |
| **140** | Serine protease HTRA1 n=1 Tax=Homo sapiens RepID=HTRA1_HUMAN | Q92743 | HTRA1 |
| **141** | Active regulator of SIRT1 n=3 Tax=Homininae RepID=AROS_HUMAN | Q86WX3 | RPS19BP1 |
| **142** | Transcription factor MafF n=2 Tax=Homo sapiens RepID=MAFF_HUMAN | Q9ULX9 | MAFF |
| **143** | DNA-directed RNA polymerase I subunit RPA43 n=1 Tax=Homo sapiens RepID=RPA43_HUMAN | Q3B726 | TWISTNB |
| **144** | Target of EGR1 protein 1 n=1 Tax=Homo sapiens RepID=TOE1_HUMAN | Q96GM8 | TOE1 |
| **145** | Protein kinase C delta-binding protein n=1 Tax=Homo sapiens RepID=PRDBP_HUMAN | Q969G5 | CAVIN3 |
|  |  |  |  |
